# A Rare Case of Cerebral Vasospasm Secondary to Primary Intraventricular Hemorrhage With an Immediate Improvement in Neurological Status Following Intra-arterial Therapy

**DOI:** 10.7759/cureus.25697

**Published:** 2022-06-06

**Authors:** Adam Delora, Rime Ezzeldin, Yazan Alderazi, Dewey Le, Mohamad Ezzeldin

**Affiliations:** 1 Department of Emergency Medicine, Hospital Corporation of America (HCA) Houston/University of Houston College of Medicine, Houston, USA; 2 Department of Medicine, Jordan University of Science and Technology, Irbid, JOR; 3 Department of Neuroendovascular Surgery, Hospital Corporation of America (HCA) Houston/University of Houston College of Medicine, Houston, USA; 4 Department of Neurology, Hospital Corporation of America (HCA) Houston/University of Houston College of Medicine, Houston, USA

**Keywords:** cerebral vasospasm, intra-arterial therapy, primary intraventricular hemorrhage, intraventricular hemorrhage, delayed cerebral ischemia, vasospasm

## Abstract

Symptomatic vasospasm following aneurysmal subarachnoid hemorrhage (SAH) occurs in roughly 30% of cases. However, vasospasm after primary intraventricular hemorrhage (IVH) is rare and described in only a handful of case reports and small retrospective studies. We present a patient with primary IVH. A conventional cerebral angiogram ruled out vascular anomalies but demonstrated severe diffuse cerebral vasospasm. The patient was treated with intra-arterial vasodilators, resulting in an immediate and profound improvement in the patient’s neurological examination. Several days later, the patient had another decline in neurological status that immediately resolved after treatment with intra-arterial therapy. To our knowledge, this is the first reported case of a profound and immediate improvement in neurological examination following intra-arterial vasodilator administration.

## Introduction

In patients who survive the initial subarachnoid hemorrhage (SAH), symptomatic vasospasm occurs in roughly 30% of patients, and angiographic vasospasm occurs in roughly 70% of patients. Most commonly, it occurs in the range of 3-14 days after the initial hemorrhage [1]. Many definitions of delayed cerebral ischemia (DCI) have been proposed, but a commonly accepted definition is “the occurrence of focal neurological impairment (such as hemiparesis, aphasia, apraxia, hemianopia, or neglect) or a decrease of at least 2 points on the Glasgow Coma Scale (GCS). This should last for at least one hour, is not apparent immediately after aneurysm occlusion, and cannot be attributed to other causes by means of clinical assessment, CT or MRI scanning of the brain, and appropriate laboratory studies” [2].

Although this phenomenon is well described in SAH, it is less common in patients with intraventricular hemorrhage (IVH). Our literature search returned 12 case reports with 14 cases of symptomatic vasospasm attributed to IVH [3-14]. Two of those cases had SAH in the basal cisterns [14]. There was a retrospective review of 36 patients with IVH diagnosed with ruptured cerebral arteriovenous malformation (AVM) who underwent a subsequent cerebral angiography. The study showed a statistically significant relationship between isolated IVH, vasospasm, and DCI development [15].

Nimodipine is a calcium channel blocker used to prevent the effects of DCI. It has been shown to decrease the incidence of poor neurological outcomes at three months (class IA). The mechanism of this medication is unclear. It does not affect angiographic vasospasm but may have some effect on the smaller vessels. Perhaps nimodipine regulates cellular calcium channel shifts that result in cell death [1]. The treatment of cerebral vasospasm includes hemodynamic augmentation by raising the mean arterial pressure (MAP) to maintain adequate cerebral perfusion pressure. This is accomplished by maintaining euvolemia and administering vasopressors. Other treatments include balloon angioplasty and intra-arterial administration of vasodilators. These vasodilators include verapamil and milrinone. Vasospasm may recur [16,17].

To our knowledge, we are reporting for the first time a patient with severely altered mentation secondary to diffuse cerebral vasospasm as a result of primary IVH who had a profound and immediate improvement in neurological function following intra-arterial vasodilator therapy.

## Case presentation

**Figure 1:**
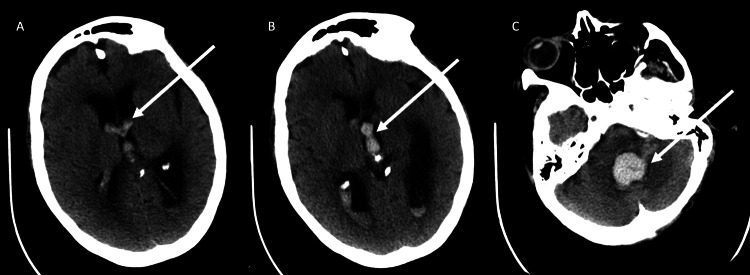
Axial cuts, non-contrast CT of the head showing intraventricular hemorrhage. (A) IVH in the fourth ventricle. (B and C) IVH and hydrocephalus in the third and bilateral lateral ventricles. White arrows point out IVH.

A 52-year-old right-handed female with a past medical history significant for liver cirrhosis, chronic systolic heart failure, depression on mirtazapine, generalized edema, chronic back pain with a long history of opiate use, deep vein thrombosis, and pulmonary embolism on apixaban arrived at the emergency room via EMS after her daughter found her unable to get out of bed and unable to follow commands. The last time the patient was at her baseline was roughly 17 hours prior. During her initial neurological examination, she withdrew to painful stimuli in both upper extremities and grimaced to painful stimuli in both lower extremities but did not open her eyes or follow commands. The patient was intubated in the emergency room for altered mental status with a GCS of 6. Vital signs included a blood pressure of 121/69 mmHg, a pulse of 78 per minute, and a temperature of 36.7°C. Ammonia was mildly elevated at 38 umol/L with a reference range of 9-30 umol/L. The patient was on methadone, but there were no signs on the physical examination of an opiate toxidrome. Her thyroid-stimulating hormone level was within the normal range. Figure 1 shows a non-contrast CT of the head with IVH and mild ventriculomegaly of the fourth ventricle. CT angiogram (CTA) of the head and neck was unremarkable. There was no clear evidence of vasospasm, aneurysm, or vascular anomaly. The patient was given prothrombin complex concentrate (PCC), and a right frontal external ventricular drain (EVD) was placed due to hydrocephalus.

The patient was monitored in the neurological ICU. Transcranial Doppler could not be obtained due to poor transtemporal acoustic windows. On day 3, she was off sedation and remained stuporous. Other etiologies of altered mentation such as sepsis, hypercarbia, and hypoglycemia were ruled out. Seizures were unlikely secondary to primary IVH [18]. The EVD was properly placed, remained functional, was open at 5 cmH20, and draining at roughly 10 cc/hour. Intracranial pressure (ICP) values were less than 15 mmHg.

**Figure 2:**
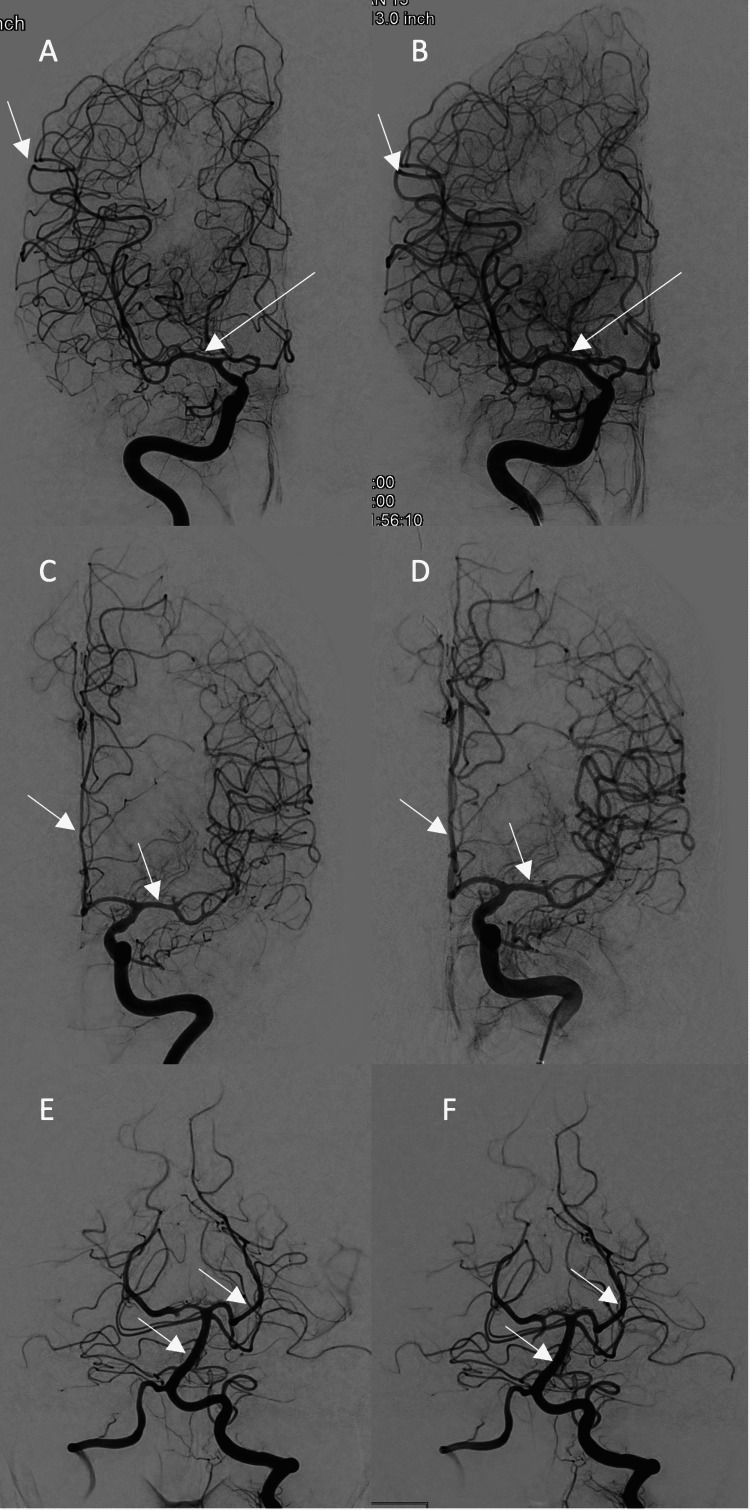
Conventional cerebral angiogram, AP view, of the bilateral internal carotid and left vertebral arteries before and after intra-arterial therapy. (A) AP view of the right internal carotid artery demonstrating severe diffuse vasospasm of the anterior, middle, and posterior cerebral artery branches. (B) AP view of the right internal carotid artery demonstrating significant improvement in the previously seen vasospasm. (C) AP view of the left internal carotid artery demonstrating severe diffuse vasospasm of the anterior and middle cerebral artery branches. (D) AP view of the left internal carotid artery demonstrating significant improvement in the previously seen vasospasm. (E) AP view of the left vertebral artery demonstrating mild diffuse vasospasm of the basilar artery and its branches. (F) AP view of the left vertebral artery demonstrating improvement in the previously seen vasospasm. White arrows point out prominent areas of vasospasm and recovery in the left and right panels, respectively.

A conventional cerebral angiogram ruled out aneurysm, vascular anomalies, and the typical beading pattern that is usually seen with reversible cerebral vasoconstriction syndrome. Angiogram demonstrated severe diffuse intracranial vasospasm of primarily the anterior circulation as shown in Figure 2A, 2C. Figure 2E shows mild vasospasm in the left vertebral artery. Cerebral vasospasm was successfully treated with intra-arterial injections of 10 mg of verapamil and 4 mg of milrinone in each of the following vessels: the right internal carotid artery (ICA) (Figure 2B), left ICA (Figure 2D), and left vertebral artery (Figure 2F), with a significant radiological improvement of the vasospasm. The patient experienced an immediate clinical improvement in her neurological examination while she was still on the angiography suite table. Her eyes spontaneously opened, she was mouthing words, and she was asking appropriate questions and following simple commands. The patient remained intubated in an attempt to optimize respiratory status.

**Figure 3:**
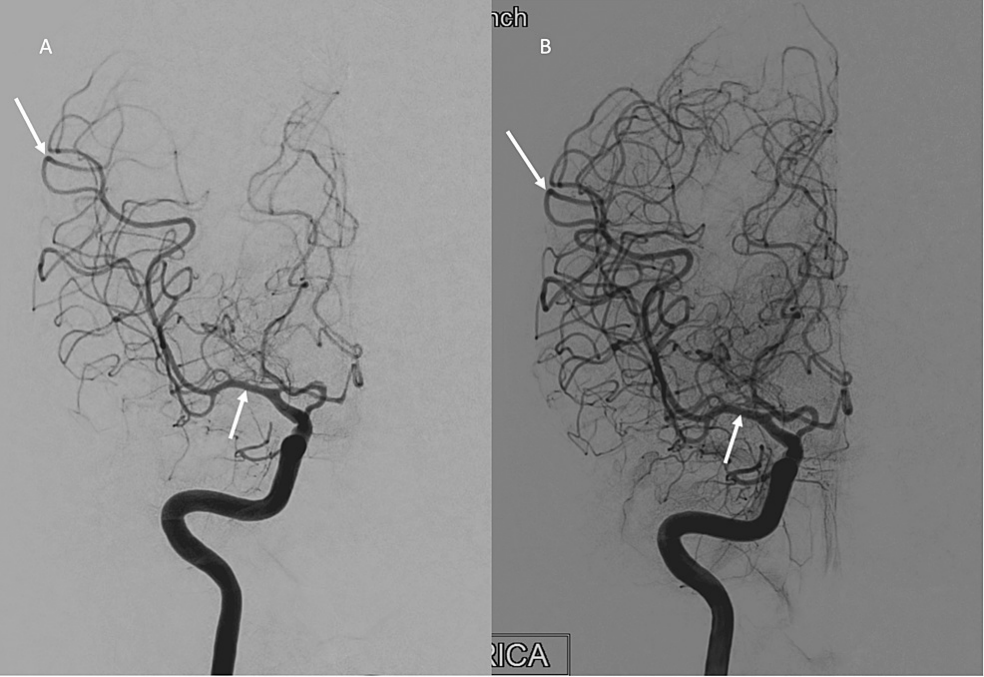
Conventional cerebral angiogram showing resolution of the second episode of vasospasm. (A) AP view of the right internal carotid artery demonstrating severe diffuse vasospasm of the anterior, middle, and posterior cerebral artery branches. (B) AP view of the right internal carotid artery demonstrating significant improvement in the previously seen vasospasm. White arrows point out prominent areas of vasospasm and recovery in the left and right panels, respectively.

On day 5, the patient became stuporous again. Vital signs included a blood pressure of 126/91 mmHg, a pulse of 65 per minute, and a temperature of 36.6°C. Repeat CT of the head was stable. The etiology of this repeated decline in her neurological status was attributed to the recurrence of the severe cerebral vasospasm. Figure 3A shows a repeat conventional cerebral angiogram demonstrating severe diffuse vasospasm of the right ICA branches. There was also mild diffuse vasospasm in the left ICA and basilar artery. The vasospasm was successfully treated with intra-arterial injection of verapamil 10 mg in each of the bilateral ICAs and right vertebral artery, and an injection of milrinone 4 mg in the right ICA. Figure 3B shows the right ICA conventional cerebral angiogram with a resolution of the cerebral vasospasm. The patient had similar prompt improvement in the radiological and clinical cerebral vasospasm and was again awake and following simple commands.

She was extubated on day 7. The patient’s neurological examination was non-focal and grossly unremarkable. She was downgraded to the intermediate care unit the next day. The patient remained in the hospital for pneumonia treatment and was subsequently discharged to rehabilitation.

## Discussion

Primary IVH is a hemorrhage in the ventricles that does not have an identifiable parenchymal origin. The terms primary and isolated IVH are used interchangeably in the literature. Secondary IVH originates from the brain parenchyma or subarachnoid space. While symptomatic vasospasm occurs in roughly 30% of SAH patients, primary IVH is only described in a handful of cases. Our literature search returned 12 case reports with 14 cases of this phenomenon. All except one of these cases involved an identifiable arteriovenous malformation (AVM) [3-14]. Two of those cases had SAH in the basal cisterns [14]. In a retrospective study that looked at 36 patients with intracranial hemorrhage diagnosed with ruptured cerebral AVM who underwent a subsequent cerebral angiography for vasospasm, there was a statistically significant relationship between primary IVH, vasospasm, and the development of DCI [15].

In one case report, the authors hypothesized that vasospasm after IVH may be overlooked because patients with IVH are not routinely examined by conventional cerebral angiography [8]. Although the incidence appears to be much lower than SAH, vasospasm after IVH may be more common than initially thought. Researchers using animal models injected autologous blood into the ventricles. These studies showed vasospasm and, on electron microscopy, showed blood elements infiltrated into the vascular cell walls [19]. The exact mechanism for vasospasm after primary IVH has not been determined, but it has been hypothesized that vasospasm secondary to primary IVH involves a constant recirculation of heme products into the subarachnoid space with subsequent propagation of cell-mediated responses [9]. In SAH, secondary IVH is an independent predictor of symptomatic vasospasm used in the Fisher and modified Fisher Scales [20]. Together, all of this research indicates that there is an association between IVH and vasospasm.

The treatments for vasospasm used in IVH come from SAH research. This is because of the higher incidence. One strategy for treating vasospasm is hemodynamic augmentation, which includes vasopressors for increasing MAP and fluids to maintain euvolemia. Intra-arterial infusion of vasodilators is also referred to as chemical angioplasty. Papaverine is a phosphodiesterase inhibitor and was the first medication to be used in chemical angioplasty. Papaverine has a poor side effect profile and can depress brainstem function and cause respiratory distress. Calcium channel blockers with better side effect profiles such as nimodipine, nicardipine, and verapamil have also been used. Milrinone is a phosphodiesterase type III inhibitor that causes vasodilation and increased inotropy. This is favorable in vasospasm when trying to increase cerebral perfusion pressure that is dependent on mean arterial pressure [12,16,17]. Balloon angioplasty has also been used to treat vasospasm in IVH [6].

To our knowledge, we are reporting for the first time a patient with cerebral vasospasm secondary to primary IVH who had a profound and immediate improvement in neurological status following intra-arterial therapy.

## Conclusions

Primary IVH resulting in cerebral vasospasm is rare and has only been reported in a few case reports. To our knowledge, this is the first reported case of a profound and immediate improvement in neurological examination following intra-arterial vasodilator administration. This case serves to add to the existing literature on IVH-related vasospasm and treatments. Further research should be done on the incidence of symptomatic vasospasm secondary to primary IVH and its response to intra-arterial vasodilator administration.
